# Supplementary material for: Probing the ultrafast dynamics of excitons in single semiconducting carbon nanotubes
Source: Nat Commun. 2022 Oct 21;13:6290. doi: 10.1038/s41467-022-33941-2 (PMC9586955; doi:10.1038/s41467-022-33941-2)
Supplement: Supplementary file 1 — Supplementary Information [file 41467_2022_33941_MOESM1_ESM.pdf]

Supplementary Information: Probing the Ultrafast Dynamics of Excitons in Single Semiconducting Carbon Nanotubes

Konrad Birkmeier,[†,¶] Tobias Hertel,[‡] and Achim Hartschuh[*,†]

†*Department of Chemistry and CeNS, LMU Munich, Butenandtstr. 5-13, 81377 Munich, Germany*

‡*Institute of Physical and Theoretical Chemistry, Julius-Maximilian University Würzburg, 97074 Würzburg, Germany*

¶*TOPTICA Photonics AG, Lochhamer Schlag 19, 82166 Gräfelfing, Germany*

E-mail: achim.hartschuh@lmu.de

Table of content

- Supplementary note 1: Experimental setup
- Supplementary note 2: Elastic scattering image
- Supplementary note 3: TiSCAT signal amplitude scaling with excitation and probe power
- Supplementary note 4: Additional TiSCAT and PL images of (6,5) and (6,4) SWCNTs
- Supplementary note 5: Fitting procedure for TiSCAT and PL transients
- Supplementary note 6: Histogram of exponential exciton decay times
- Supplementary note 7: Number of initially created excitons
- Supplementary note 8: Monte-Carlo simulations of exciton decay
- Supplementary references

Supplementary note 1: Experimental setup

The experimental setup used for detecting TiSCAT and time-resolved PL signals from semiconducting (6,5) SWCNTs on glass substrates combines a pulsed laser system with a scanning confocal optical microscope (Fig. 1). A tunable Ti:Sa laser produces $\approx$ 150 fs pulses at a rate of 76 MHz which are split into pump and probe beams by a beam splitter (BS). For PL measurements, the probe beam is blocked. The probe pulse frequency ω_{probe} for TiSCAT experiments is obtained by pumping a white-light generating photonic crystal fiber (PCF) followed by spectral filtering using a narrow bandpass filter (bandwidth 10 nm). The intensity of the pump pulses at the laser output ω_{pump} is modulated by an acousto optical modulator (AOM) at a frequency of 96 kHz serving as the reference for the lock-in amplifier. The pump pulses are then sent to an optical delay stage to control the time-delay Δt and recombined with the probe pulses using a beam splitter. The colinear beams are reflected by a beam splitter and focused onto the sample using a microscope objective with high numerical aperture (NA = 1.49) to form a tight, diffraction-limited spot. The reflected and scattered light is collected by the same objective. A longpass filter is used to suppress the pump-light. For TiSCAT, the light is guided to a sensitive photodiode (PD). The PD signal output is connected to a lock-in amplifier synchronized with the AOM signal to allow for signal demodulation at the first harmonic. In the case of time-resolved PL experiments, the light is reflected by a flip mirror (FM) towards a single-photon counting avalanche photodiode (APD) connected to time-correlated single-photon counting (TCSPC) electronics. For recording PL spectra, the light is sent to a spectrometer equipped with a sensitive charge coupled device (CCD) camera.

For the TiSCAT measurements on (6,4) SWCNTs included in this supplementary material (Fig. 5 and 6) we utilized a tunable dual wavelength fiber laser with improved stability for excitation at 780 nm and probing at 880 nm, respectively. Unfortunately, this very stable laser system cannot be tuned to 1000 nm, the probe wavelength of (6,5) nanotubes.

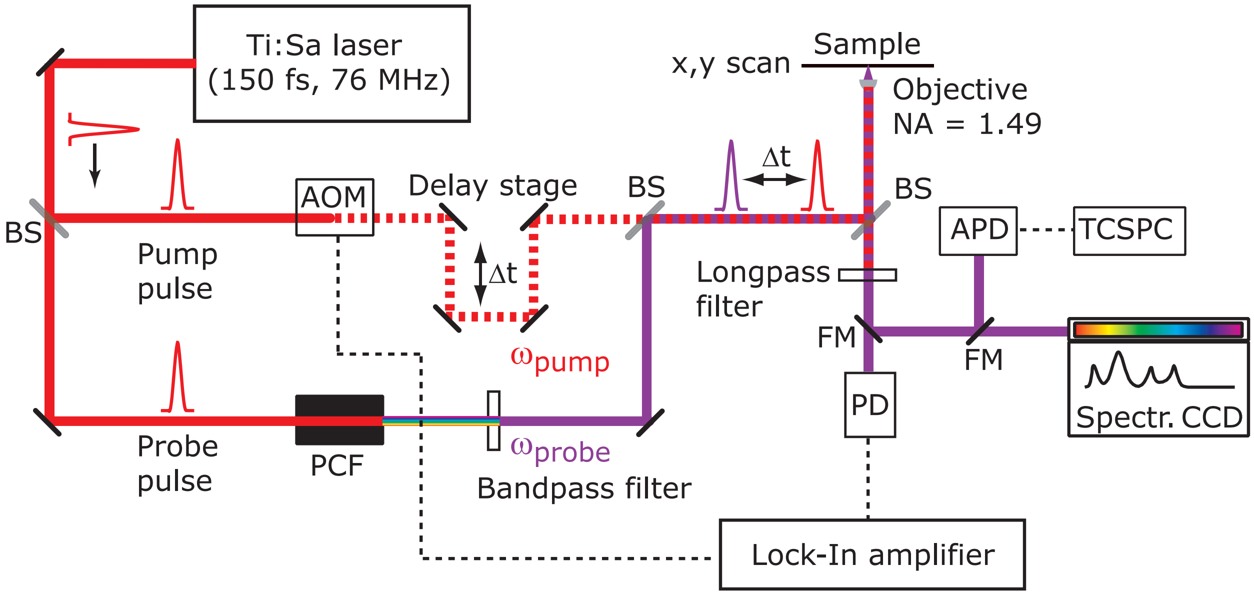

Supplementary Figure 1: **Experimental setup for detecting TiSCAT and time-resolved PL signal from single semiconducting SWCNTs.** BS: Beamsplitter, AOM: Acousto optical modulator, PCF: Photonic crystal fiber, FM: Flip mirror, PD: Photodiode, APD: Avalanche photodiode for single photon detection, TCSPC: Time-correlated single photon counting electronics, NA: Numerical aperture of microscope objective.

Supplementary note 2: Elastic scattering image

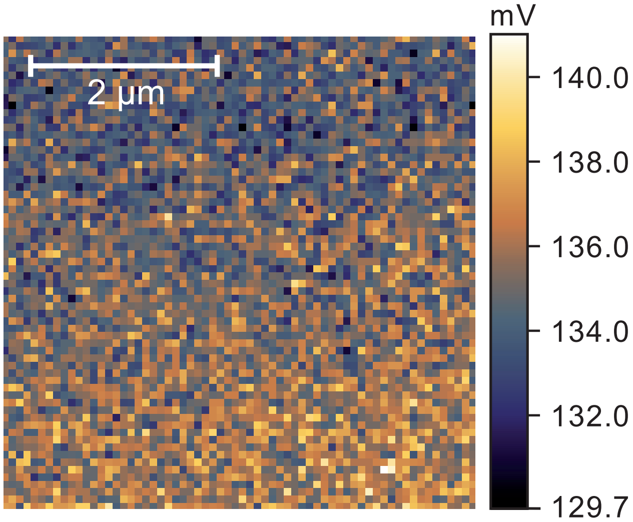

Supplementary Figure 2: **Elastic scattering image recorded simultaneously with the TiSCAT image shown in Fig. 1c of the main manuscript.** Besides laser stability, elastic scattering at a single detection energy lacks the specificity needed to identify SWCNTs. This means that SWCNTs could not be distinguished from other scattering objects, such as possible dust particles or residual surfactant. In the present experiment this specificity is obtained by detecting the pump-induced changes.

Supplementary note 3: TiSCAT signal amplitude scaling with excitation and probe power

Linear scaling of the transient absorption signal at zero delay time was observed for the range of pump and probe powers used in the present experiments (Fig. 3a,b). Absorption saturation at both pump and probe energies can therefore be neglected in the present experiments.

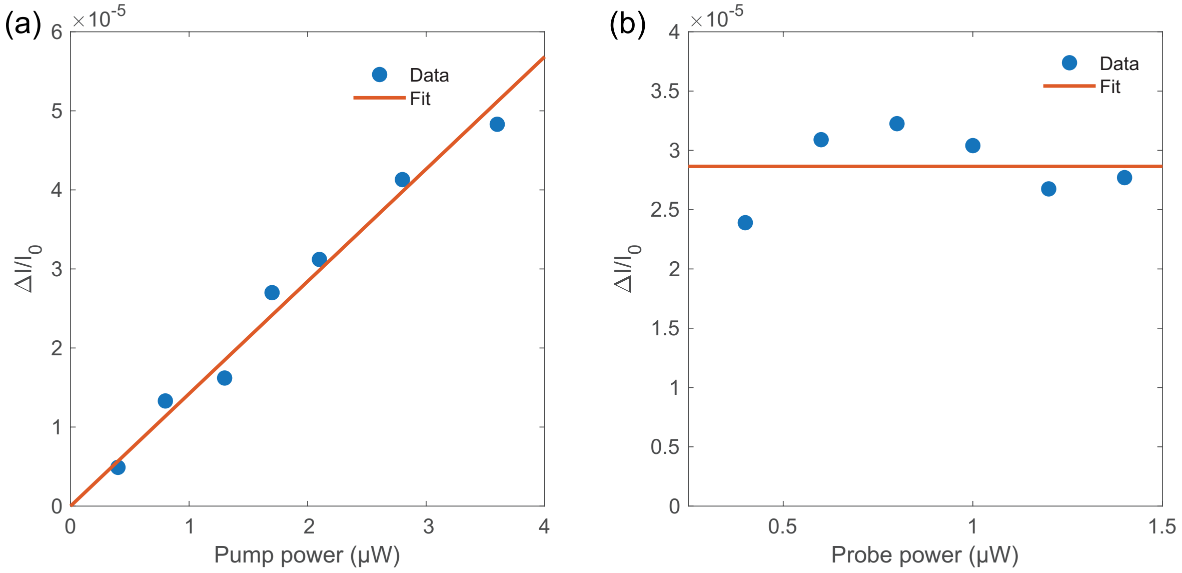

Supplementary Figure 3: **Power dependence of the TiSCAT signal. a** TiSCAT signal amplitude detected for a single (6,5) SWCNT at zero delay time ($\Delta t = 0$) upon increasing the excitation power. Linear scaling is observed showing that absorption saturation can be neglected for the power range used in the present study. **b** TiSCAT signal amplitude detected for a single (6,5) SWCNT upon increasing the probe power.

Supplementary note 4: Additional TiSCAT and PL data

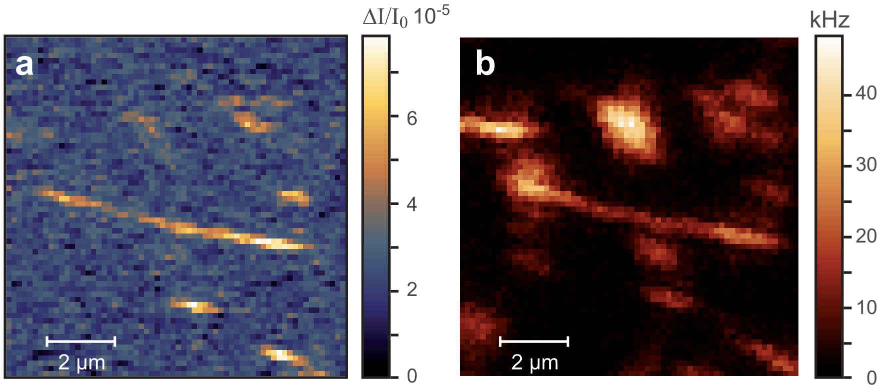

Supplementary Figure 4: **Confocal transient interferometric scattering (a) and PL image (b) of (6,5) SWCNTs on glass.** For both images the nanotubes were excited at 880 nm and $2.3 \cdot 10^5$ photons per pulse. The transient interferometric scattering image was detected at zero delay between pump and probe pulse ($\Delta t = 0$) at a probing wavelength of 1000 nm with $6.5 \cdot 10^4$ photons per pulse.

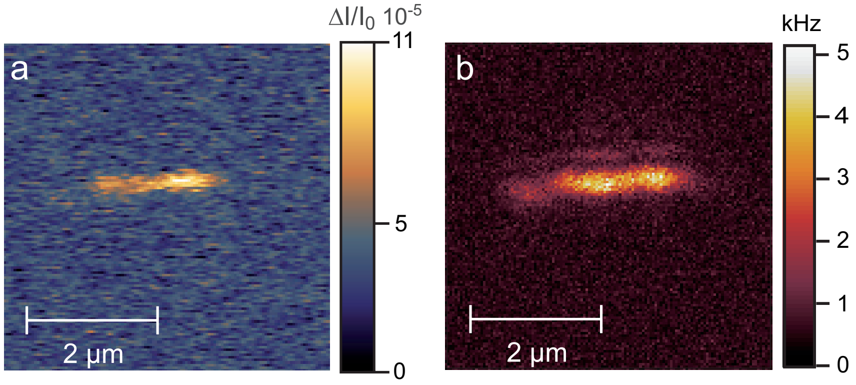

Supplementary Figure 5: **Confocal transient interferometric scattering (a) and PL image (b) of a (6,4) SWCNT on glass.** For both images the nanotubes were excited at 780 nm and $1.4 \cdot 10^5$ photons per pulse. The transient interferometric scattering image was detected at zero delay between pump and probe pulse ($\Delta t = 0$) at a probing wavelength of 880 nm with $7.7 \cdot 10^4$ photons per pulse.

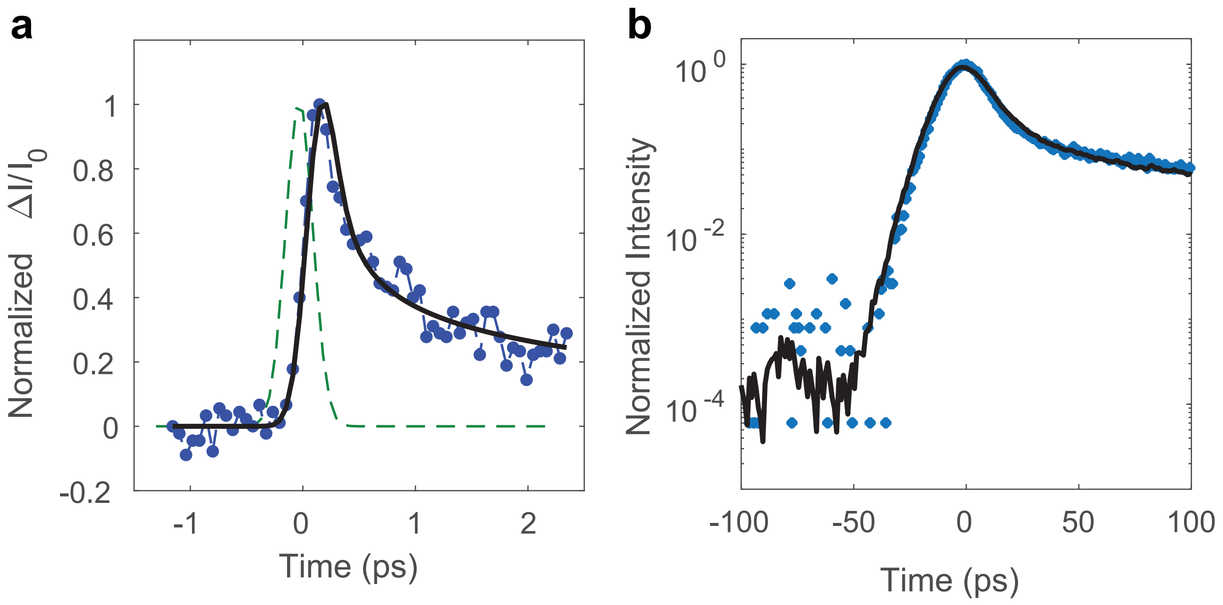

Supplementary Figure 6: **Exciton dynamics in a single (6,4)-SWCNT. a** Pump-probe transient detected at a pump wavelength of 780 nm with $1.4 \cdot 10^5$ photons per pulse. The probe wavelength was centered at 880 nm, which is in resonance with the E_{11} transition of the (6,4) SWCNT. The corresponding fit-function given by eqn. 3 in the main manuscript is depicted as solid line. **b** The PL transient of the investigated SWCNT at the same excitation conditions can be described by the model function (solid line) with the identical exponential exciton lifetime as for the pump-probe measurement. The slower signal component is mainly due to the instrument response function (see fig. 2c in the main text for comparison).

Supplementary note 5: Fitting procedure for TiSCAT and PL transients

For all investigated nanotubes both types of transients were fitted by the same model function defined in eqn. 3. To account for the different instrument response functions (IRF) of the techniques, the fitted model functions were convoluted with the independently measured IRFs. In the case of time-resolved PL measurement the IRF was determined by detecting the elastically scattered light from the sub-ps probe pulse at 1000 nm, the wavelength of PL emission. For the IRF of the TiSCAT experiments, we used the measured width of the sum-frequency generation signal between pump- and probe pulse on iron-iodate nanocrystals.[1] For all nanotubes we first fitted the PL transient to determine the slower exponential decay time as well as a first estimate of the faster diffusional time. We then used these parameters to fit the TiSCAT transient in the first few ps keeping the slower exponential decay time fixed. We then fixed the diffusional time and optimized the exponential decay using the PL transient and repeated this procedure until the parameters converged.

Supplementary note 6: Histogram of exponential exciton decay times

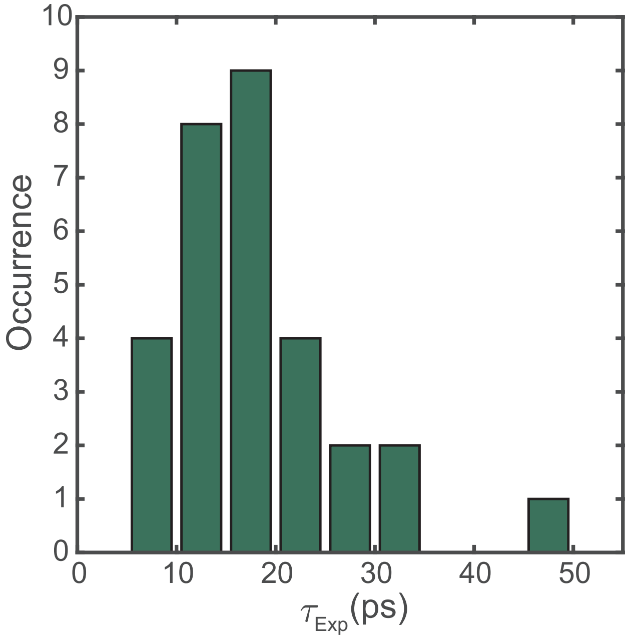

Supplementary Figure 7: **Histogram of the determined exponential decay times τ_{exp}.** The observed exponential lifetimes will strongly be influenced by the defect density as well as the occurence and efficiency of exciton localization in a given nanotube.[2–5]

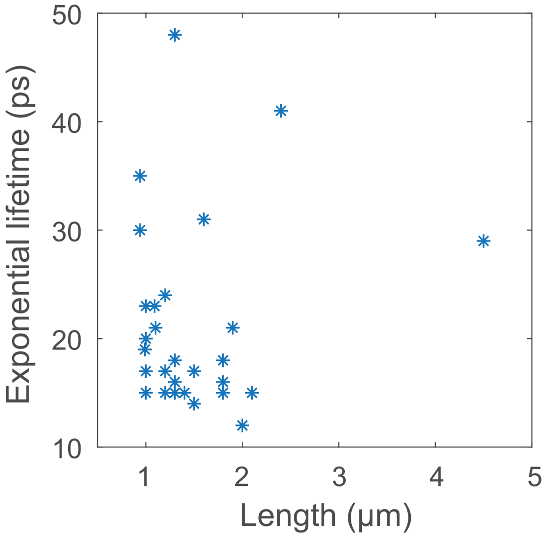

Supplementary Figure 8: **Exponential lifetimes vs. nanotube lengths**. The PL dynamics of 28 single (6,5) SWCNTs were investigated and their exponential exciton lifetime was determined as described in the main manuscript. Their length was determined from the corresponding confocal PL images. Whereas exciton quenching at the nanotube ends would be expected to lead to shorter lifetimes for shorter nanotubes, no clear correlation can be observed here. We explain this as follows: First, all transients were detected in the center of the nanotubes reducing the influence of end-quenching. Second, the diffraction-limited width of pump-and probe focii results in spatial averaging on a length scale of about 300 nm. This will smear out finite length effects also from short nanotubes. Third, the observed exponential lifetimes will strongly be influenced by the defect density as well as the occurence and efficiency of exciton localization in a given nanotube as mentioned above.

Supplementary note 7: Number of initially created excitons

The number of incident photons per pulse $N_{photons}$ can be connected to the number of initially created excitons $N_{excitons}$ using the absorption cross section σ of (6,5) SWCNTs. Here we take the value $\sigma = 3.2 \cdot 10^{-17}\mathrm{cm}^2/\mathrm{C}$ atom from ref. 6 measured at the E_{22} transition (561 nm) for single (6,5) SWCNTs deposited on glass substrates with light polarized along the nanotube axis in the focus of a high NA microscope objective closely matching the experimental conditions of this study. In the present experiment, the SWCNTs are excited off-resonance at 880 nm with approx. 3 times smaller absorption efficiency as determined from the ensemble absorption spectrum in Fig. 1e of the main manuscript corresponding to an absorption cross section per nm of $\sigma = 8.6 \cdot 10^{-16}\ \mathrm{cm}^2\mathrm{nm}^{-1}$. The total absorption cross section per nanotube then amounts to σL_{CNT}. Here, the excitation focus diameter d_f is significantly smaller than the nanotube length (Fig. 1c of the main manuscript) such that the actual absorption cross section corresponds to σd_f The diffraction-limited focus diameter is given by the $d_f = 1.22\lambda/NA = 720\ \mathrm{nm}$. With a focus area of $A_f = \pi(d_f/2)^2$ the absorption probability α becomes $\alpha = \sigma d_f/A_f = 1.6 \cdot 10^{-4}$. This value applies to the case of plane wave excitation and will be slightly smaller for strongly focused excitation due to the appearance of orthogonal polarization components.[7] In the experiment, nanotubes oriented mostly parallel to the polarization direction of the excitation and probing field that render the strongest optical response where chosen.

The number of initially created excitons then is $N_{excitons} = \alpha N_{photons}$ (Fig. 3b of the main manuscript). From this we can calculate the average exciton-exciton distance $d_0 = d_f/(2N_{excitons})$ (Ref. 8), where we consider a statistical Gaussian distribution of excitons following the spatial profile of the focused excitation pulse.

Supplementary note 8: Monte-Carlo simulations of exciton decay

The Monte-Carlo simulation of the exciton dynamics is initialized by distributing the excitons along the one-dimensional nanotube divided into segments determined by the step size and the length of the tube (see below). The number of initially created excitons is chosen as described in sec. . The individual exciton position is assigned by a random number generation which follows a Gaussian probability distribution. The exciton distribution depicted in Fig. 9 shows the sum of 75.000 exciton position allocations and fits well with the theoretically expected Gaussian shape of the excitation focus spot geometry rendered as red line.

After the initial distribution of the excitons their stepwise movement is simulated under the initial assumption of an identical probability for a step to one or the other direction. This probability for an exciton movement is determined by the mean squared displacement and the spatial step size of the simulation. After each temporal iteration step the distance between the individual excitons along the carbon nanotube is computed and pairs of excitons whose spacing is equal or smaller than the defined exciton-exciton-annihilation distance (EEA distance) are removed from the simulation and do not interact in the further iterations. As a second decay mechanism each single exciton may decay independently after each step representing a mono-exponential decay process. The probability for this decay type is determined by the exponential lifetime and the temporal step size of the simulation. The third decay path is given by the end quenching mechanism, which applies in the simulation in the case of an exciton reaching one end of the simulated nanotube.

After each temporal step, the number of remaining excitons is counted and stored. The whole simulation process is iteratively repeated and the temporal transient is extracted as the temporal evolution of the mean exciton number. The red data curve in Fig. 10 shows a typical transient computed from the Monte-Carlo simulation described above which fits well to eqn. 3 (black line) introduced in the main manuscript. To account for a localization site a single Gaussian-shaped energy minimum of the exciton binding energy is implemented in the simulation at the centre of the nanotube. Based on the description in ref. 9 the probability for a step to the right or the left is therefore spatially modified. The resulting decay dynamics showed a significant speed up of

the exciton-exciton annihilation, which can be seen in Fig. 11 as blue data curve and which is discussed in the main manuscript.

Numerical parameters:

- Step size: 1 nm
- Time step: 0.1 fs
- Intrinsic lifetime (exponential lifetime): 10 ps
- Focus width (Airy unit): 721 nm
- Length of SWCNT: 1 μm
- Number of iterations: 15 000
- EEA distance: 5 nm (EEA occurs for distances $\leq$ 5 nm)
- Trap position: Center of SWCNT
- Trap depth: 50 meV
- Width of trap (Gaussian width): 20 nm

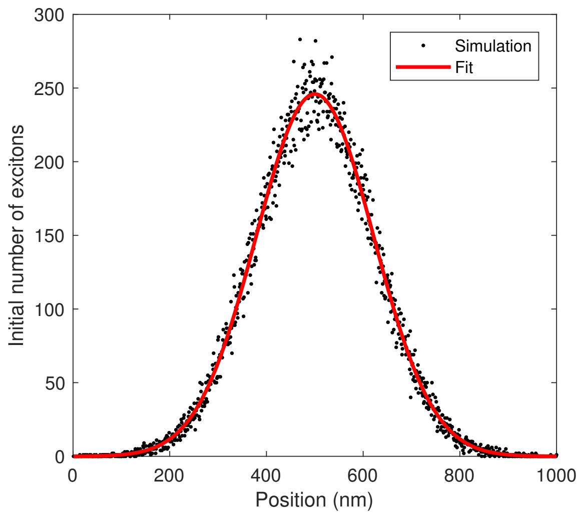

Supplementary Figure 9: Total initial exciton distribution used in a Monte-Carlo simulation. The spatial distribution reflects the intensity profile of the focused pump laser beam.

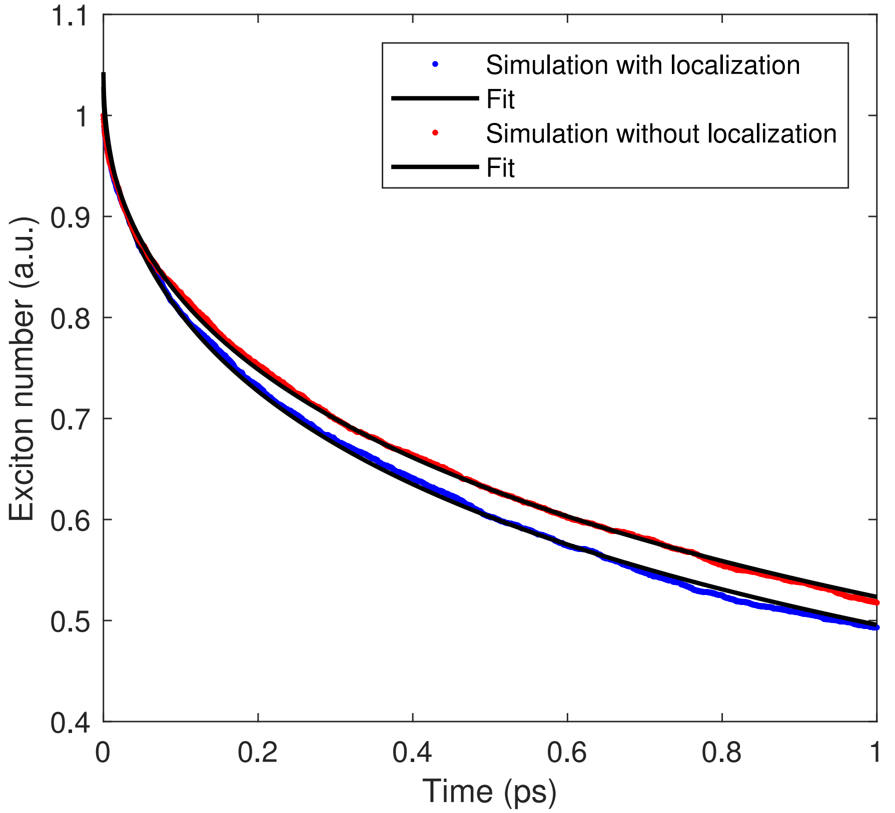

Supplementary Figure 10: Simulated transient exciton population for a single SWCNT (L = 2 μm) excited in the center with and without exciton localization within the first ps after pulsed excitation (initial number of excitons $N_0 = 5$. Exciton localization was induced by implementing a single Gaussian-shaped energy minimum with a depth of 50 meV and a width of 20 nm in the center of the nanotube (see Fig. 11). Exciton localization is seen to significantly speed up exciton-exciton annihilation in this configuration. Also shown are the fitted model functions using eqn. 3 from the main manuscript from which the diffusion times τ_D shown in Fig. 5(b) (main manuscript) are derived.

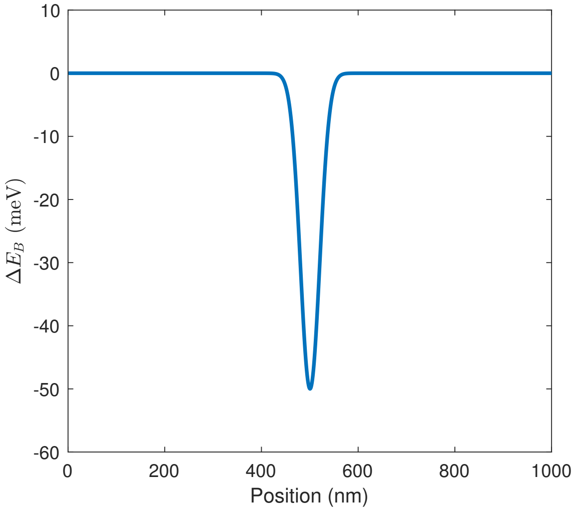

Supplementary Figure 11: Exciton energy landscape used to describe a single localization site. The localization site was modelled as a Gaussian-shaped energy minimum $\propto -A\exp(-x^2/w^2)$ with depth A = 50 meV and width w= 20 nm.

Supplementary References

(1) Bonacina, L.; Mugnier, Y.; Courvoisier, F.; Le Dantec, R.; Extermann, J.; Lambert, Y.; Boutou, V.; Galez, C.; Wolf, J. . *Appl. Phys. B: Laser Opt.* **2007**, *87*, 399.

(2) Hagen, A.; Steiner, M.; Raschke, M. B.; Lienau, C.; Hertel, T.; Qian, H.; Meixner, A. J.; Hartschuh, A. Exponential decay lifetimes of excitons in individual single-walled carbon nanotubes. *Phys. Rev. Lett.* **2005**, *95*, 197401–197404.

(3) Gokus, T.; Cognet, L.; Duque, J. G.; Pasquali, M.; Hartschuh, A.; Lounis, B. Mono- and biexponential luminescence decays of individual single-walled carbon nanotubes. *J. Phys. Chem. C* **2010**, *114*, 14025–14028.

(4) Duque, J. G.; Pasquali, M.; Cognet, L.; Lounis, B. Environmental and Synthesis-Dependent Luminescence Properties of Individual Single-Walled Carbon Nanotubes. *ACS Nano* **2009**, *3*, 2153–2156.

(5) Ishii, A.; Machiya, H.; Kato, Y. K. High Efficiency Dark-to-Bright Exciton Conversion in Carbon Nanotubes. *Phys. Rev. X* **2019**, *9*, 041048.

(6) Oudjedi, L.; Parra-Vasquez, A. N. G.; Godin, A. G.; Laurent, C.; Lounis, B. Metrological Investigation of the (6,5) Carbon Nanotube Absorption Cross Section. *J. Phys. Chem. Lett.* **2013**, *4*, 1460–1464.

(7) Novotny, L. and Hecht, B., *Principles of nano-optics*; Cambridge University Press: Cambridge, 2006.

(8) Srivastava, A.; Kono, J. Diffusion-limited exciton-exciton annihilation in single-walled carbon nanotubes: A time-dependent analysis. *Phys. Rev. B* **2009**, *79*, 205407.

(9) Georgi, C.; Green, A. A.; Hersam, M. C.; Hartschuh, A. Probing exciton propagation and quenching in carbon nanotubes with near-field optical microscopy. *ACS Nano* **2010**, *4*, 5914.
